# Supplementary material for: Spatial engineering of single-atom Fe adjacent to Cu-assisted nanozymes for biomimetic O2 activation
Source: Nat Commun. 2024 Mar 12;15:2239. doi: 10.1038/s41467-024-46528-w (PMC10933453; doi:10.1038/s41467-024-46528-w)
Supplement: Supplementary file 1 — Supplementary information [file 41467_2024_46528_MOESM1_ESM.pdf]

Supplementary Information

Spatial engineering of single-atom Fe adjacent to Cu-assisted nanozymes for biomimetic O_2 activation

Ying Wang[1,8], Vinod K. Paidi[2,8], Weizhen Wang[3], Yong Wang[1], Guangri Jia[4], Tingyu Yan[5], Xiaoqiang Cui[4], Songhua Cai[3,*], Jingxiang Zhao[5,*], Kug-Seung Lee[6,*], Lawrence Yoon Suk Lee[1,7,*], & Kwok-Yin Wong[1,*]

[1] State Key Laboratory of Chemical Biology and Drug Discovery, Department of Applied Biology and Chemical Technology, The Hong Kong Polytechnic University, Hung Hom, Kowloon, Hong Kong SAR, China

[2] European Synchrotron Radiation Facility, 71 Avenue des Martyrs, Grenoble, 38043 Cedex 9, France

[3] Department of Applied Physics, The Hong Kong Polytechnic University, Hung Hom, Kowloon, Hong Kong SAR, China

[4] State Key Laboratory of Automotive Simulation and Control, Department of Materials Science, Key Laboratory of Automobile Materials of MOE, Jilin University, Changchun, 130012 China

[5] Key Laboratory of Photonic and Electronic Bandgap Materials of MOE, College of Chemistry and Chemical Engineering, Harbin Normal University, Harbin 150025, PR China

[6] Pohang Accelerator Laboratory (PAL), Pohang University of Science and Technology (POSTECH), Pohang, 37673 Republic of Korea

[7] Research Institute for Smart Energy, The Hong Kong Polytechnic University, Hung Hom, Kowloon, Hong Kong SAR, China

[8] These authors contributed equally: Ying Wang, Vinod K. Paidi

[*] Corresponding emails: songhua.cai@polyu.edu.hk (S. Cai); xjz_hmily@163.com (J. Zhao); lks3006@postech.ac.kr (K.-S. Lee); lawrence.ys.lee@polyu.edu.hk (L.Y.S. Lee); kwok-yin.wong@polyu.edu.hk (K.-Y. Wong)

Supplementary Figures

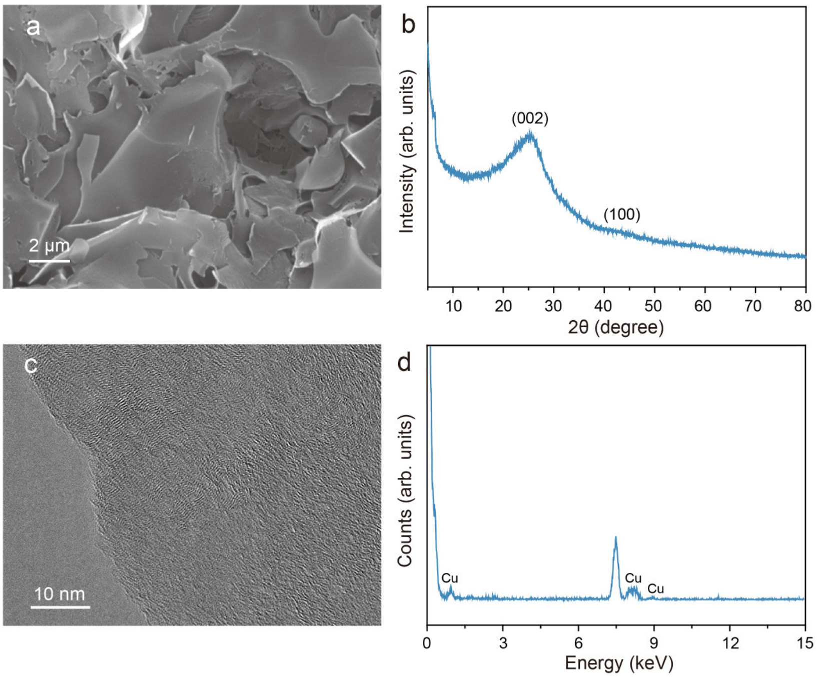

Supplementary Fig. 1. a–d, SEM image (**a**) (n = 3 images from three independent samples), XRD pattern (**b**), HRTEM image (**c**) (n = 3 images from three independent samples), and EDS spectrum (**d**) of 2D-Cu–N–C. Source data are provided as a Source Data file.

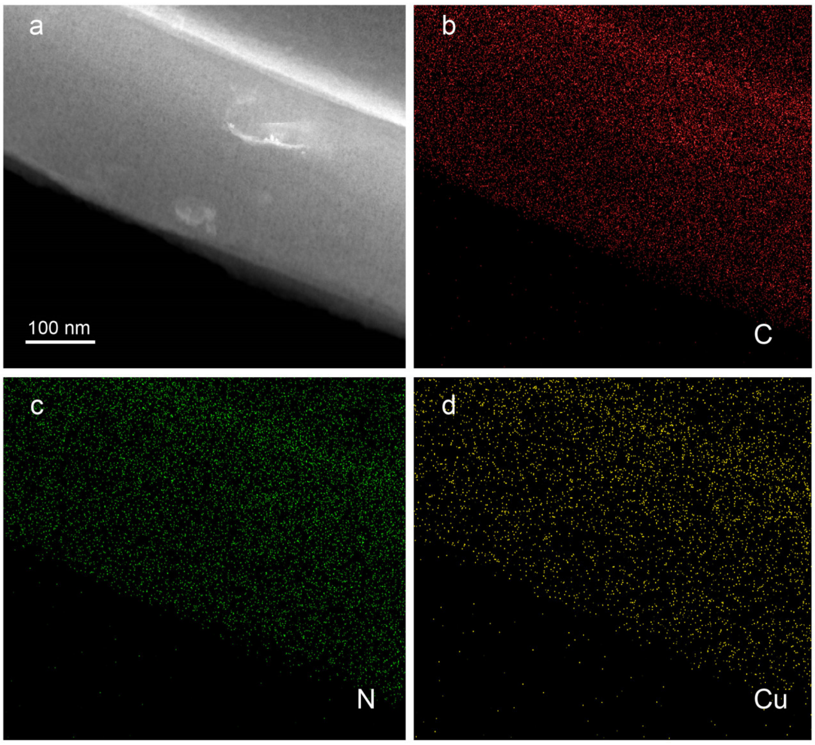

Supplementary Fig. 2. a–d, HAADF-STEM image (**a**) of 2D-Cu–N–C and the corresponding energy-dispersive X-ray (EDX) mapping images for C (**b**, red), N (**c**, green), and Cu (**d**, yellow) elements (*n* = 3 images from three independent samples).

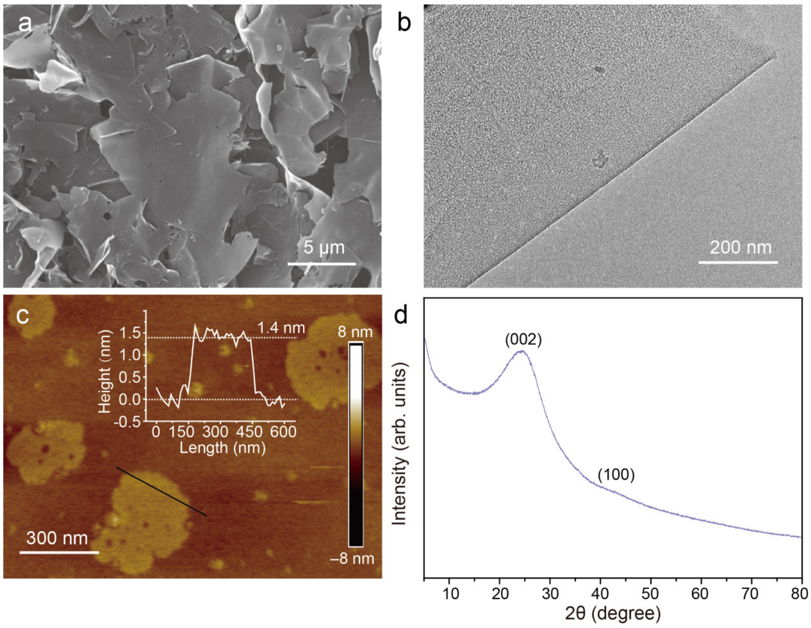

Supplementary Fig. 3. a–d, SEM (**a**), TEM (**b**), AFM images (**c**) (n = 3 images from three independent samples), and XRD pattern (**d**) of 2D-Fe–N–C. Source data are provided as a Source Data file.

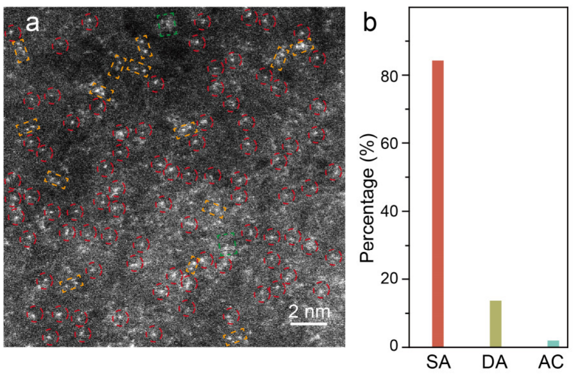

Supplementary Fig. 4. a, HAADF-STEM image of 2D-Fe–N–C (n = 3 images from three independent samples) and **b**, the corresponding statistical study of the single-atom (SA, red circles), dual-atom (DA, orange rectangles), and atomic clusters (AC, green rectangles). Source data are provided as a Source Data file.

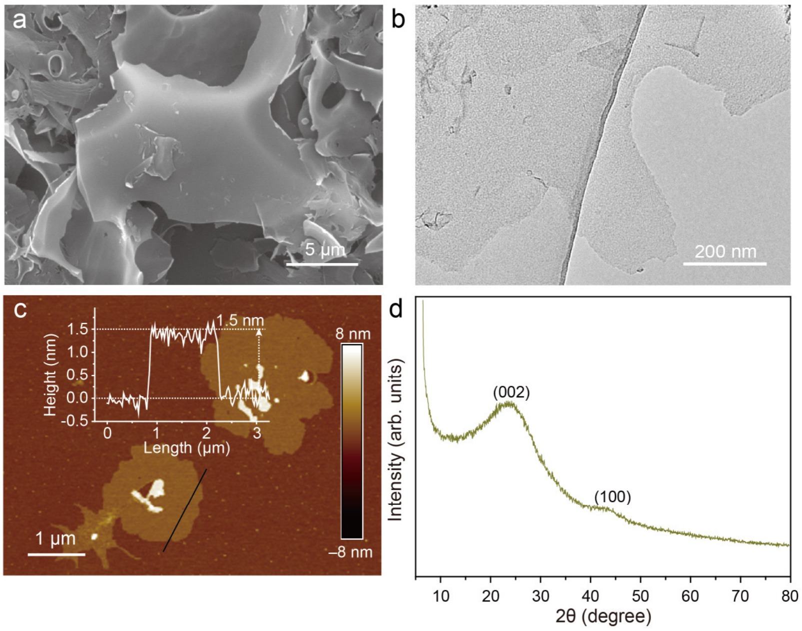

Supplementary Fig. 5. a–d, SEM (**a**), TEM (**b**), AFM images (**c**) (n = 3 images from three independent samples), and XRD pattern (**d**) of 2D-FeCu–N–C. Source data are provided as a Source Data file.

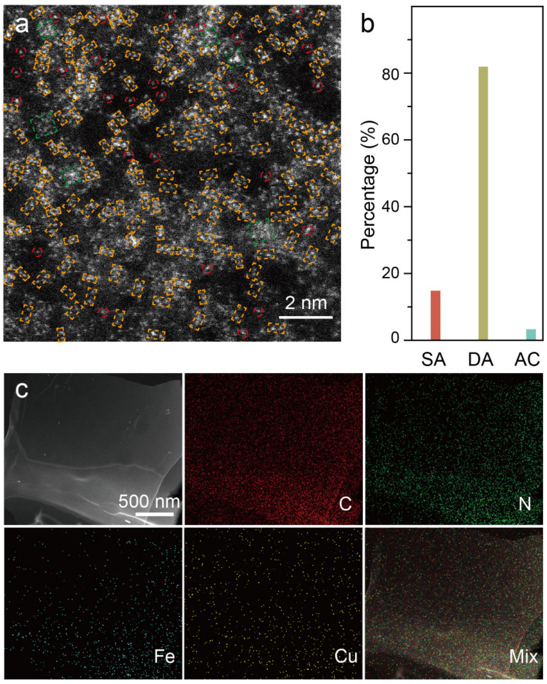

Supplementary Fig. 6. a, HAADF-STEM image of 2D-FeCu–N–C (n = 3 images from three independent samples) and **b**, the corresponding statistical study of the single-atom (SA, red circles), dual-atom (DA, orange rectangles), and atomic clusters (AC, green rectangles). **c**, HAADF-STEM image of 2D-FeCu–N–C with the corresponding C (red), N (green), Fe (blue), and Cu (yellow) elemental mapping images (n = 3 images from three independent samples). Source data are provided as a Source Data file.

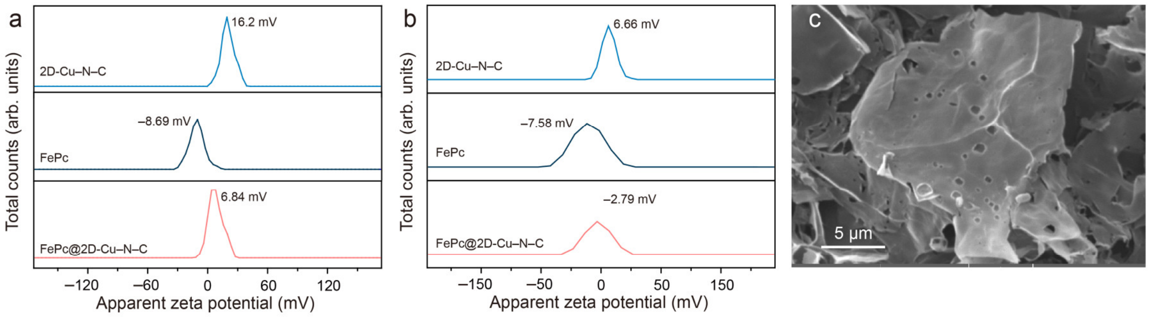

Supplementary Fig. 7. a–b, Zeta potentials of FePc, 2D-Cu–N–C, and FePc@2D-Cu–N–C in the HAc–NaAc buffer medium (pH = 4.0, **a**) and DMF medium (pH = 7.0, **b**). **c**, SEM image of FePc@2D-Cu–N–C (n = 3 images from three independent samples). Source data are provided as a Source Data file.

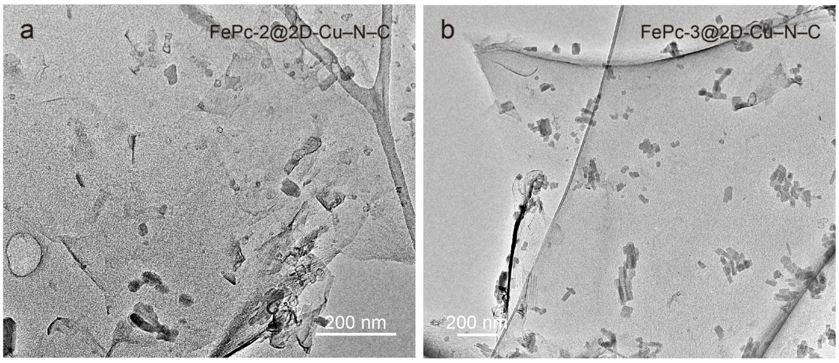

Supplementary Fig. 8. a–b, TEM images of FePc-2@2D-Cu–N–C (**a**), and FePc-3@2D-Cu–N–C (**b**) (*n* = 3 images from three independent samples).

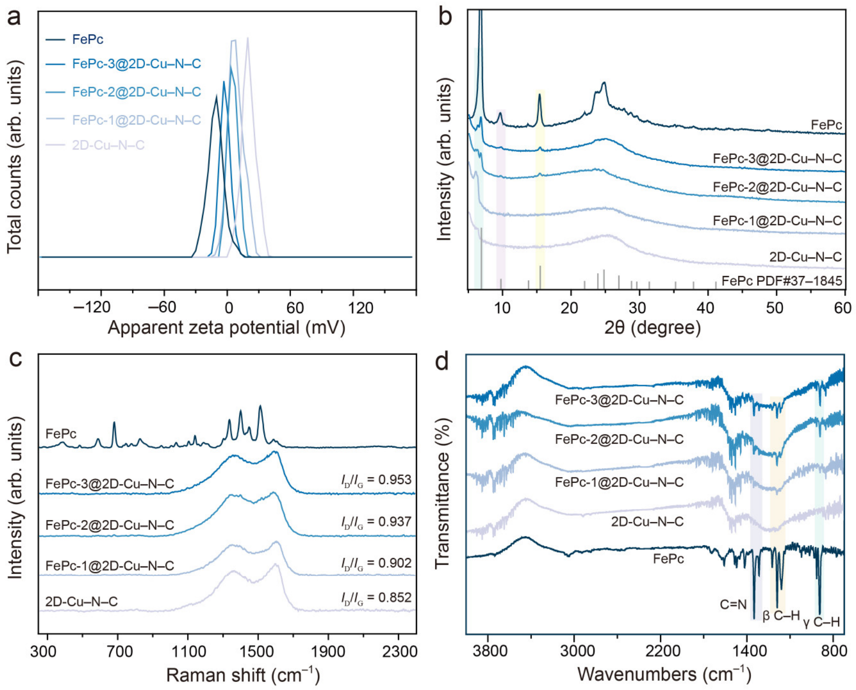

Supplementary Fig. 9. a–d, Zeta potentials in the HAc–NaAc buffer medium (pH = 4.0) (**a**), XRD patterns (**b**), Raman spectra (**c**), and FT-IR spectra (**d**) of 2D-Cu–N–C supported on various amounts of FePc. Source data are provided as a Source Data file.

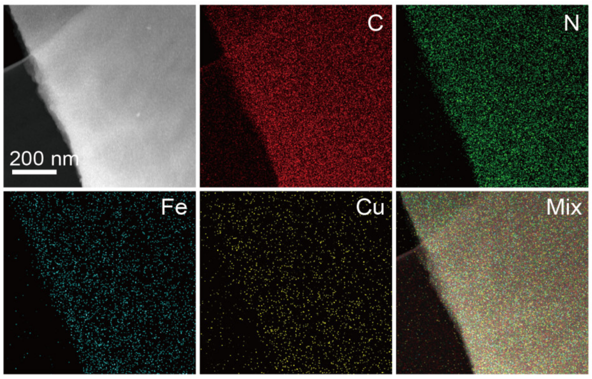

Supplementary Fig. 10. HAADF-STEM image of FePc@2D-Cu–N–C and the corresponding C (red), N (green), Cu (yellow), and Fe (blue) elemental mapping images (n = 3 images from three independent samples).

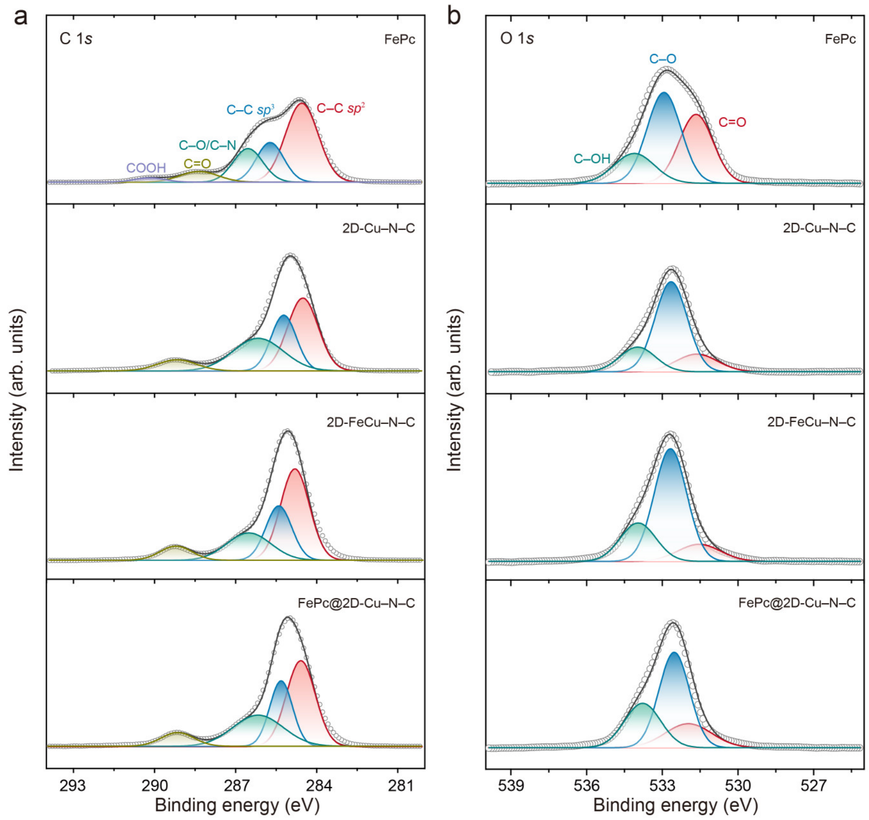

Supplementary Fig. 11. a–b, High-resolution XPS spectra of FePc, 2D-Cu–N–C, 2D-FeCu–N–C, and FePc@2D-Cu–N–C catalysts in the C 1*s* (**a**) and O 1*s* (**b**) regions. Source data are provided as a Source Data file.

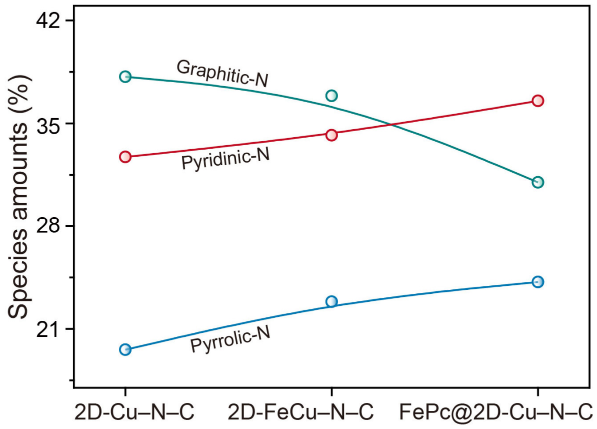

Supplementary Fig. 12. Relative population of N species in 2D-Cu–N–C, 2D-FeCu–N–C, and FePc@2D-Cu–N–C catalysts determined by XPS analysis. Source data are provided as a Source Data file.

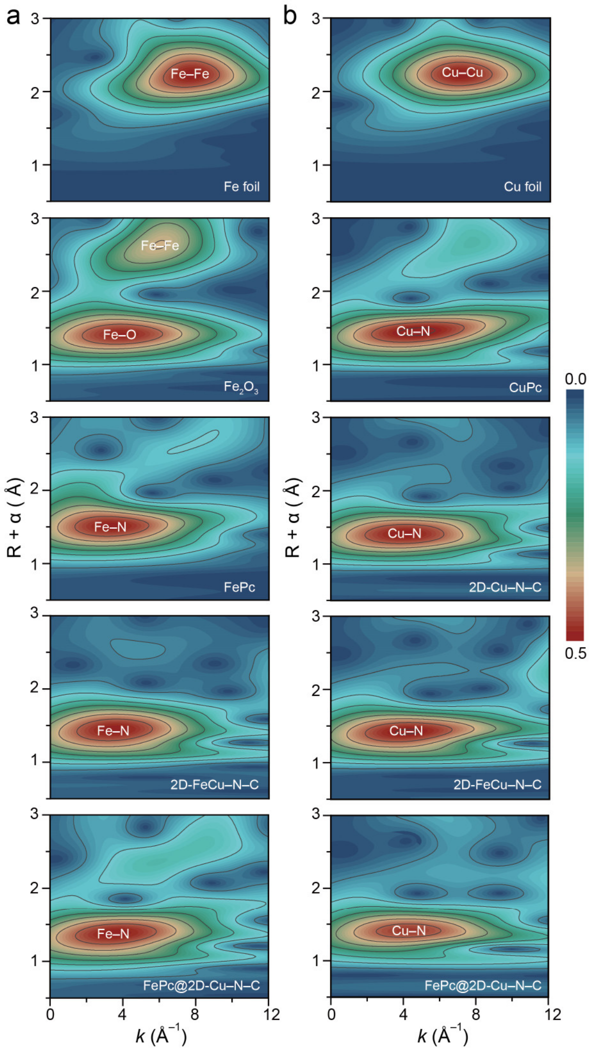

Supplementary Fig. 13. a–b, Wavelet transform of the k^2-weighted EXAFS data of Fe-based samples (**a**) and Cu-based samples (**b**).

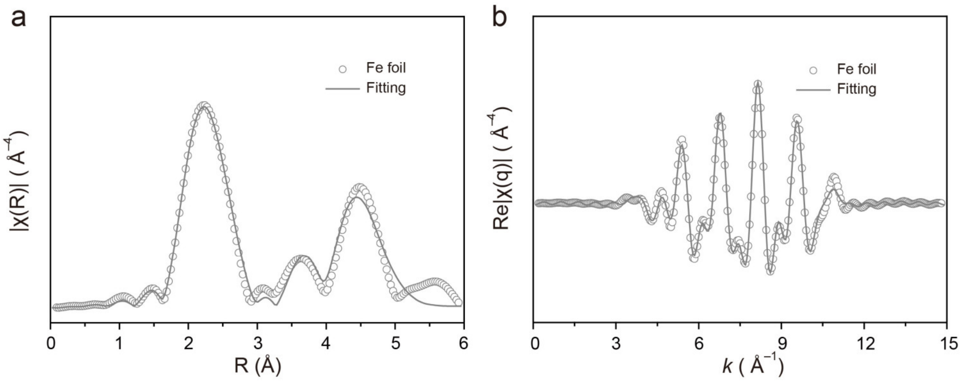

Supplementary Fig. 14. a–b, Fe K-edge EXAFS fitting analysis of Fe foil in R space (**a**) and *q* space (**b**). Source data are provided as a Source Data file.

Supplementary Note 1:

The XAS data were collected in the transmission mode for 2D-Cu–N–C, 2D-Fe–N–C, 2D-FeCu–N–C, FePc@2D-Cu–N–C, and the standard samples. The obtained spectra were processed by the ATHENA module of Demeter software packages[1, 2]. Extended X-ray absorption fine structure (EXAFS) spectra were fitted using the ARTEMIS module in a Fourier-transform range of 3–10.5 Å^{-1} with a Hanning window applied between 1.1 and 2.8 Å. The amplitude reduction factor (S_o^2) was obtained by fitting the Fe/Cu metal foil. XANES simulations of the Fe and Co K-edges were performed using the FDMNES code[3, 4]. The structural models used for the FDMNES input files were based on the DFT calculations.

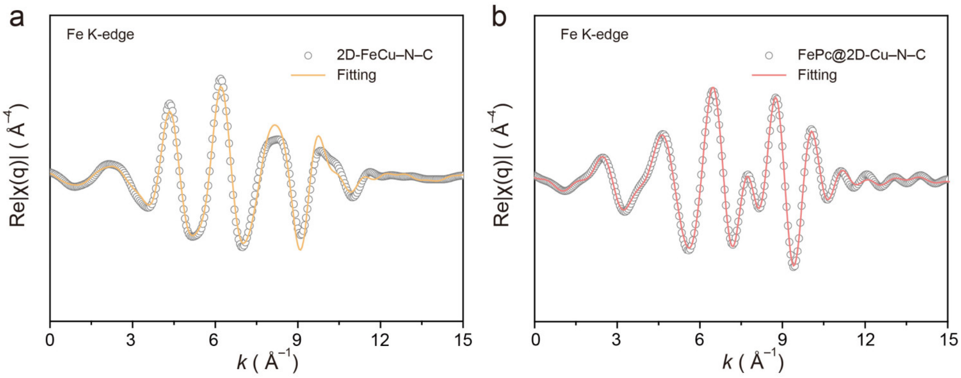

Supplementary Fig. 15. a–b, Fe K-edge EXAFS fitting analysis of 2D-FeCu–N–C (**a**) and FePc@2D-Cu–N–C (**b**) catalysts in *q* space. Source data are provided as a Source Data file.

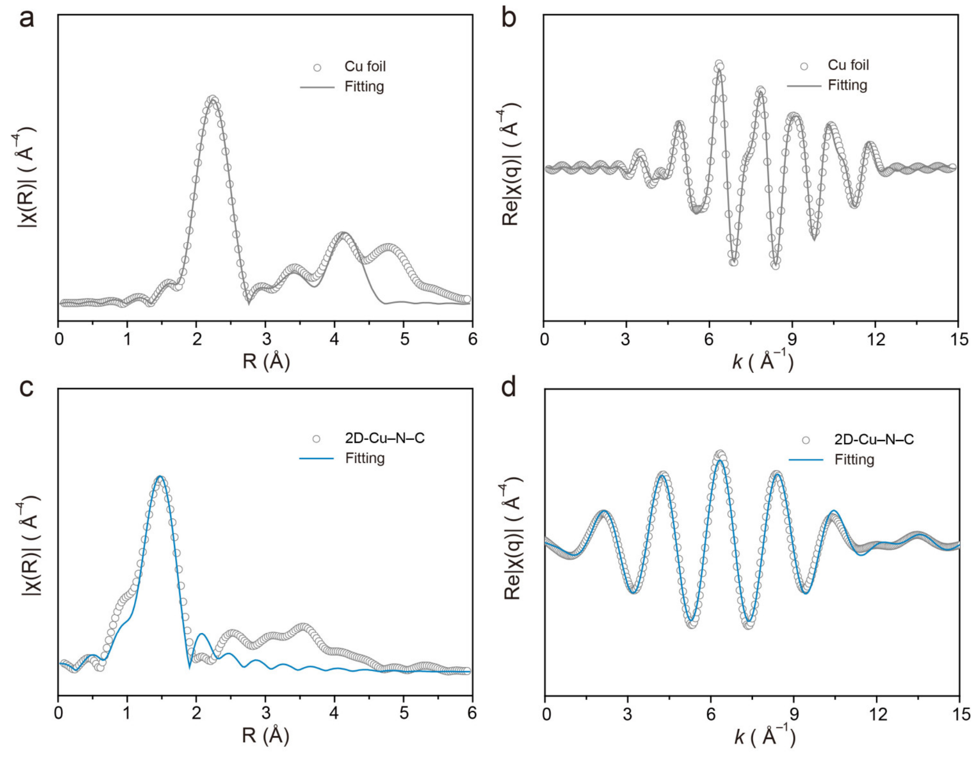

Supplementary Fig. 16. a–d, Cu K-edge EXAFS fitting analysis of Cu foil (**a, b**) and 2D-Cu–N–C (**c, d**) catalyst in R space and *q* space. Source data are provided as a Source Data file.

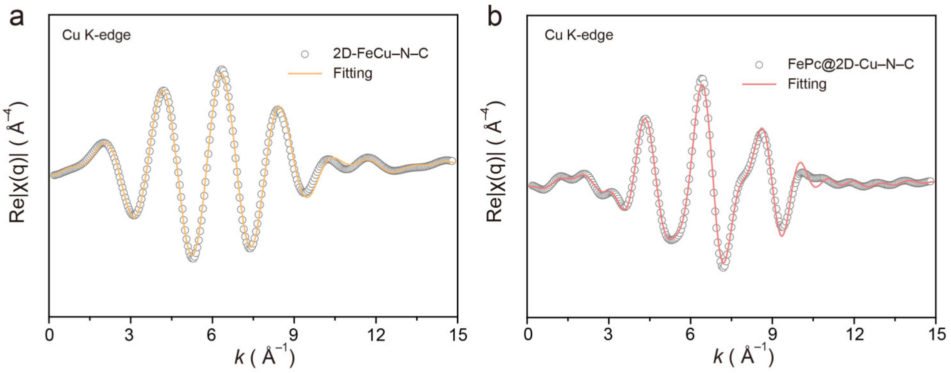

Supplementary Fig. 17. a–b, Cu K-edge EXAFS fitting analysis of 2D-Cu–N–C (**a**) and FePc@2D-Cu–N–C (**b**) catalysts in *q* space. Source data are provided as a Source Data file.

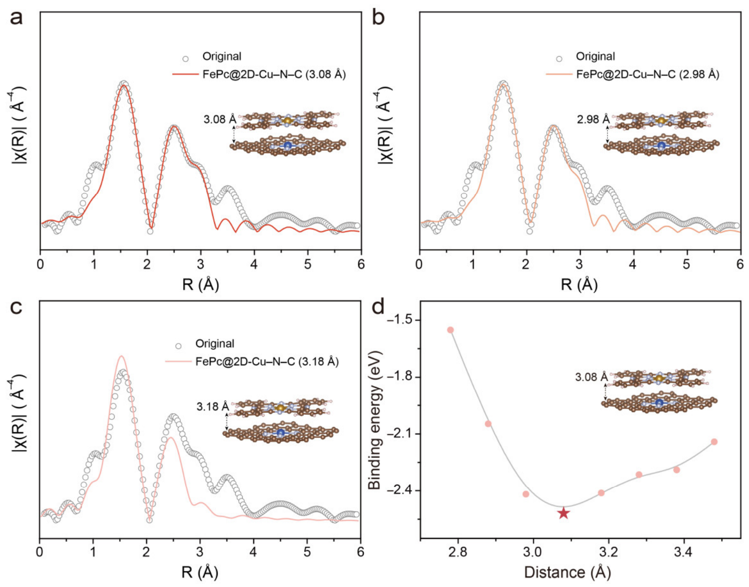

Supplementary Fig. 18. a–c, Fe K-edge EXAFS fitting analysis of FePc@2D-Cu–N–C with different layer distances in R space. **d,** The binding energy of FePc molecules and 2D-Cu–N–C supports as a function of their layer distances. Note that only the first model (a) is in good agreeement with the experiment and the other models disagree with the experiment. Source data are provided as a Source Data file.

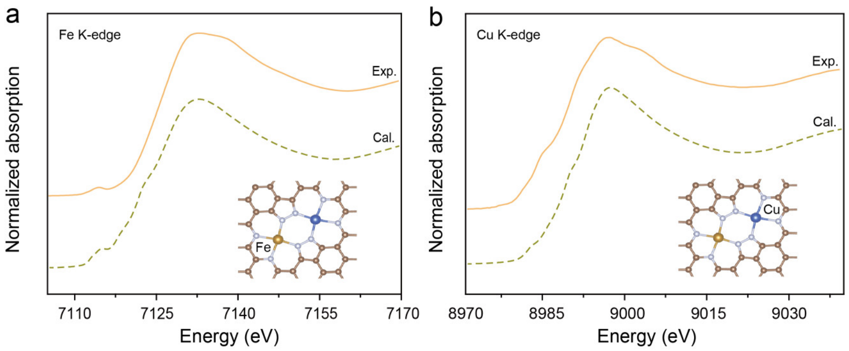

Supplementary Fig. 19. a–b, Experimental and theoretical Fe K-edge (**a**) and Cu K-edge (**b**) XANES spectra of the 2D-FeCu–N–C. Source data are provided as a Source Data file.

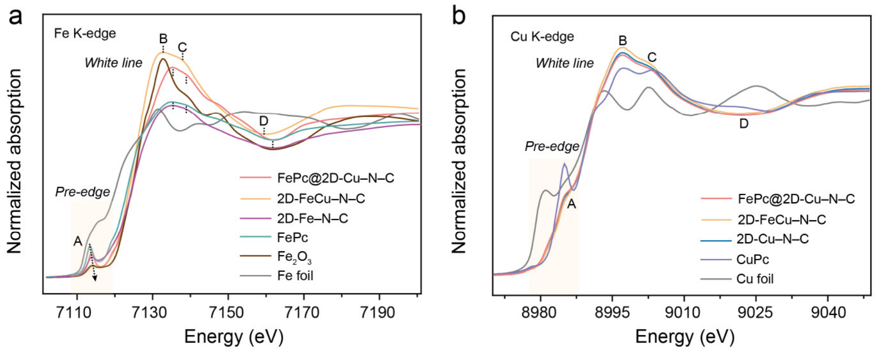

Supplementary Fig. 20. a–b, Normalized Fe K-edge (**a**) and Cu K-edge (**b**) XANES spectra of various catalysts and the corresponding reference samples. Source data are provided as a Source Data file.

Supplementary Note 2:

There are also two features (denoted as B and C) for the white line, which are attributed to the transitions of 1s → $4p_{xy}$ and multiple scattering processes, respectively (**Supplementary 20a**)[5]. The distortion degree of $Fe–N_4$ square-planarity is closely correlated to the relative intensity ratio of peaks B and C (I_B/I_C)[6]. The I_B/I_C value of the 2D-FeCu–N–C (1.025) is similar to that of FePc (1.023), which confirms its $Fe–N_4$ planarity is comparable to the rigid construction of FePc. In contrast, the FePc@2D-Cu–N–C shows an increased I_B/I_C value of 1.044, which implies that the axial FePc species bound to 2D-Cu–N–C would affect the local $Fe–N_4$ square-planarity in the FePc@2D-Cu–N–C. It is worth noting that the I_B/I_C values from the XANES spectra of the Cu K-edge are almost similar among 2D-Cu–N–C, 2D-FeCu–N–C, and FePc@2D-Cu–N–C samples (**Supplementary 20b**). It can be concluded that the $Cu–N_4$ moieties in these three samples display almost identical distortion degrees. Meanwhile, the broad feature (denoted as D) from the scattering processes is related to the threshold energy (ΔE). Based on Natoli's rule, the average Cu–N first shell bond length (d) is coupled to the value of ΔE ($\Delta E \times d^2$ = constant)[7]. The similar average Cu–N bond lengths in the three samples indicate the stable in-plane $Cu–N_4$ coordination. Thus, the negative shift in the peak D of FePc@2D-Cu–N–C suggests a longer d value than that of 2D-FeCu–N–C. In fact, the bond length of Fe–N (2.01 Å) in the 2D-FeCu–N–C is slightly longer than that of Fe–N (1.99 Å) in the FePc@2D-Cu–N–C according to the FT-EXAFS spectra of Fe K-edge.

Supplementary Note 3:

We also note that the strong interaction between the FePc molecule and 2D-Cu–N–C matrix would influence the local symmetry. The XANES feature at ~7,113 eV in FePc arises from the characteristic electric-dipole forbidden transition of 1s → 3*d* transition (**Supplementary 20a**)[7]. Similar characteristic peaks appear in the pre-edge region of 2D-FeCu–N–C and FePc@2D-Cu–N–C, verifying the existence of D_{4h} symmetry of the square planar Fe–N_4 configuration[8]. It is noted that the intensity of this peak is weaker with lower symmetry in the FePc@2D-Cu–N–C than that in FePc. This observation evidences that the square-planar Fe–N_4 moieties of the axial FePc would be influenced by the electronic interaction with the 2D-Cu–N–C plane.

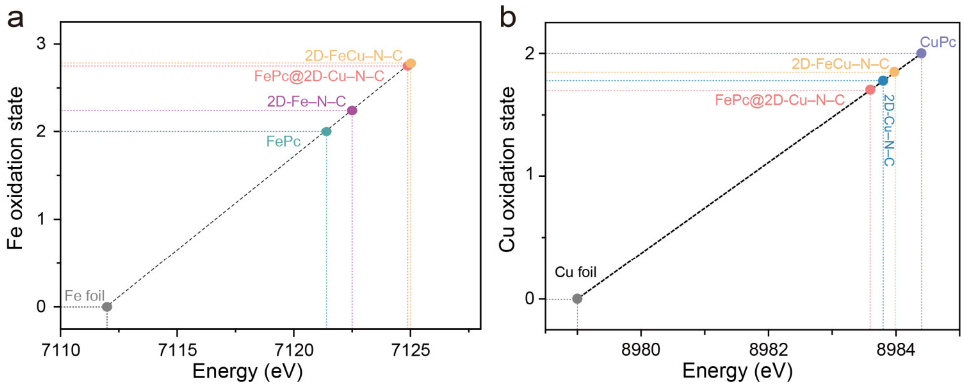

Supplementary Fig. 21. a–b Fe (**a**) and Cu (**b**) oxidation state of various catalysts plotted as a function of the pre-edge position in the Fe K-edge and Cu K-edge XANES spectra. Dashed line is the theoretical fit of the data. Source data are provided as a Source Data file.

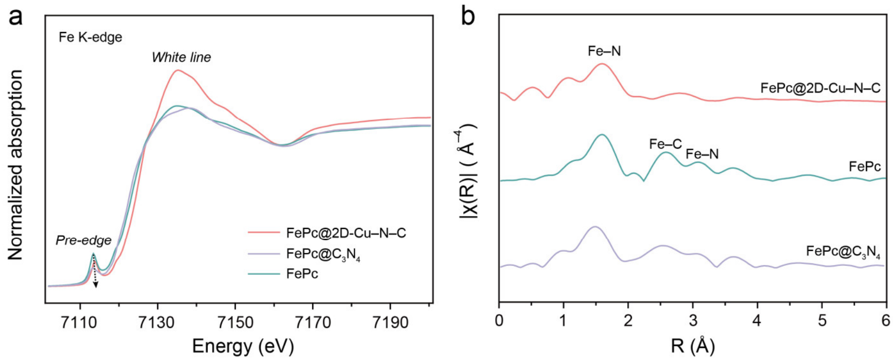

Supplementary Fig. 22. a–b, Normalized Fe K-edge XANES spectra **(a)** and the Fourier transformed magnitude of the k^3-weighted EXAFS spectra (**b**) of FePc, FePc@C_3N_4, and FePc@2D-Cu–N–C. Source data are provided as a Source Data file.

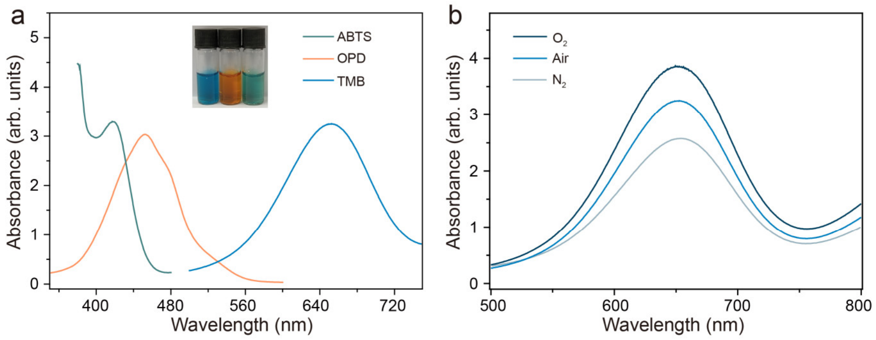

Supplementary Fig. 23. UV−vis absorption spectra of the FePc@2D-Cu–N–C-catalyzed oxidation of (**a**) typical substrates (ABTS, OPD, and TMB) and (**b**) TMB oxidation under various conditions (oxygen-saturated, air-saturated, and nitrogen-saturated HAc–NaAc buffer). Source data are provided as a Source Data file.

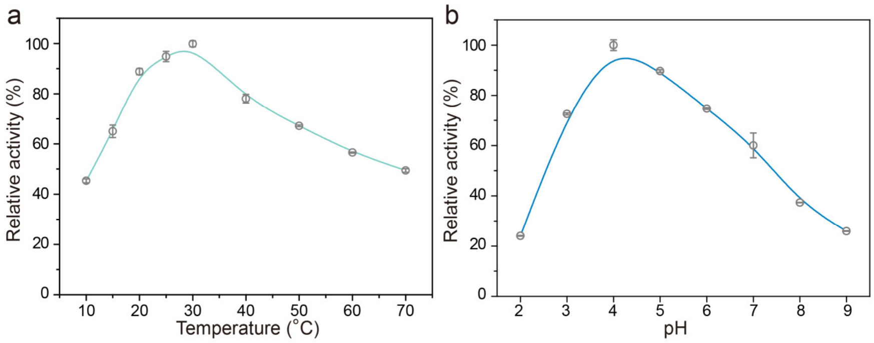

Supplementary Fig. 24. a–b, Optimization of the oxidase-like activity of FePc@2D-Cu–N–C nanozyme with the control of temperature (**a**) and pH (**b**). n = 3 independent experiments and all data are presented as mean values ± SD. Source data are provided as a Source Data file.

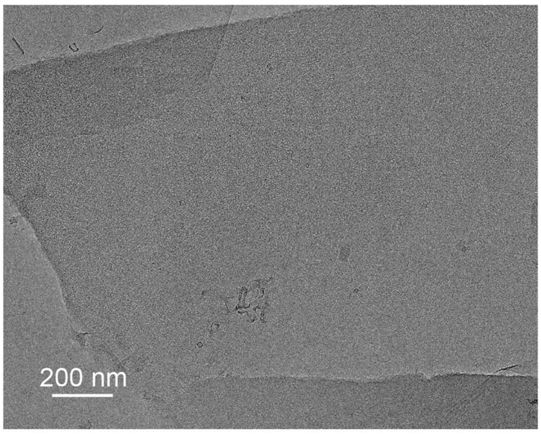

Supplementary Fig. 25. TEM image of the FePc@2D-Cu–N–C after the OXD-like reaction (n = 3 images from three independent samples).

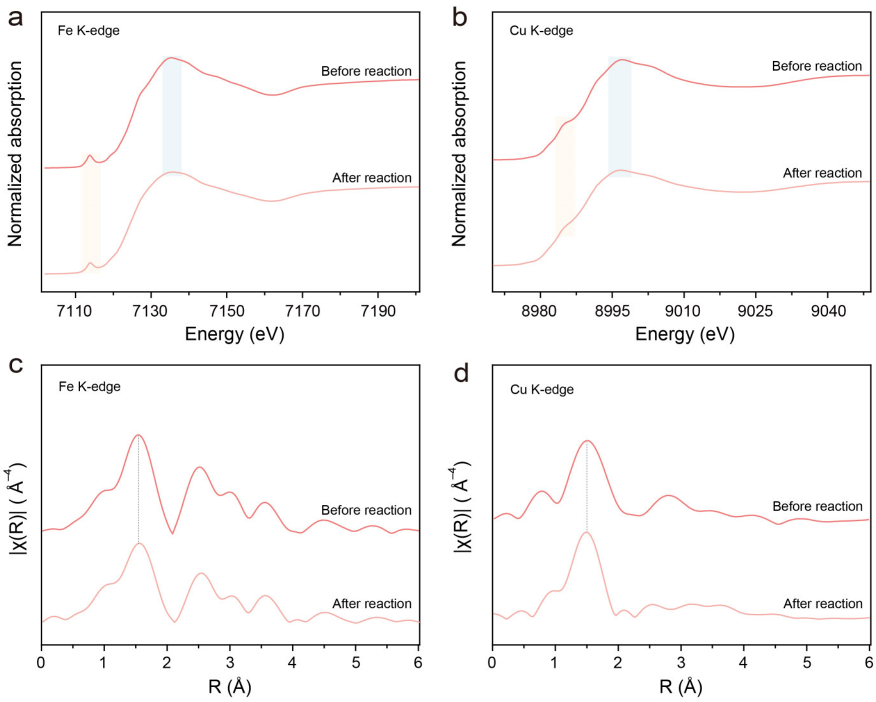

Supplementary Fig. 26. a–d, The Fe K-edge and Cu K-edge XANES spectra (**a–b**) and FT-EXAFS spectra (**c–d**) of FePc@2D-Cu–N–C before and after the OXD-like reaction. Source data are provided as a Source Data file.

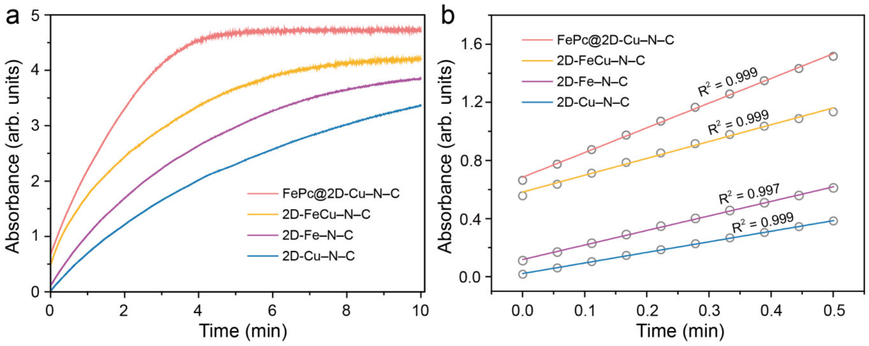

Supplementary Fig. 27. **a**, Time-dependency profiles of TMB colorimetric reaction at 652 nm catalyzed by the equal amount of 2D-Cu–N–C, 2D-Fe–N–C, 2D-FeCu–N–C, and FePc@2D-Cu–N–C nanozymes. **b**, Magnified initial linear portion of the time evolution curves of absorbance at 652 nm. A length of 30 s was chosen for the calculation of the initial rate due to the R^2 regression coefficients. Source data are provided as a Source Data file.

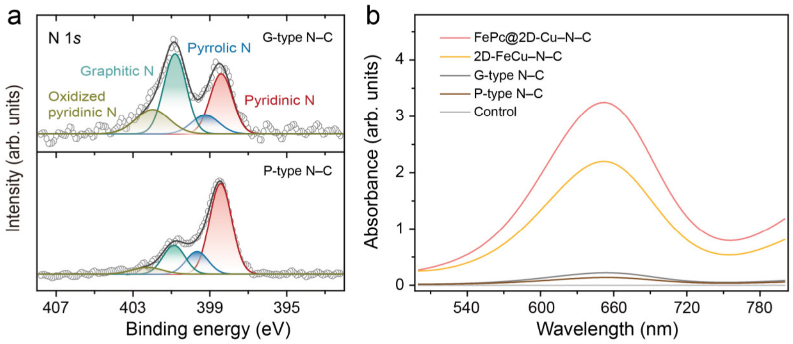

Supplementary Fig. 28. **a**, High-resolution XPS spectra in the N 1*s* regions of graphitic N-rich N–C (G-type N–C) and pyridinic N-rich N–C (P-type N–C) comparisons.. **b**, The corresponding oxidase-like activities based on the UV−vis absorption spectra of $_{ox}$TMB. Source data are provided as a Source Data file.

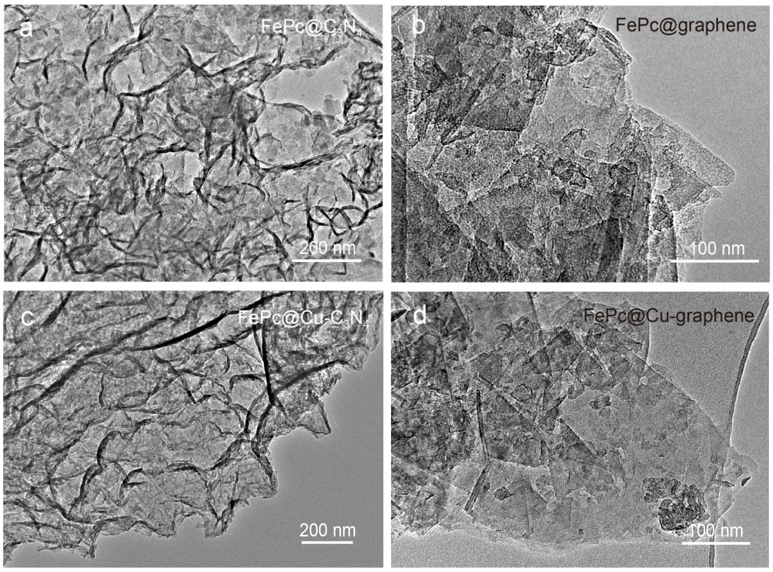

Supplementary Fig. 29. **a–d**, TEM images of FePc@C_3N_4 (**a**), FePc@graphene (**b**), FePc@Cu-C_3N_4 (**c**), and FePc@Cu-graphene (**d**) (*n* = 3 images from three independent samples).

Supplementary Note 4:

Graphitic carbon nitride (C_3N_4) was synthesized following the reported procedure[9]. In a typical process, melamine (500 mg) was transferred to an aluminum oxide ceramic boat in a tube furnace and heated to 550 °C with a ramping rate of 8 °C·min^{-1} under 5% H_2/Ar atmosphere

for 4 h. After naturally cooled down to the room temperature, the product was collected and grinded.

Cu-C_3N_4 was synthesized following the reported procedure[9]. Melamine (500 mg) and copper (II) chloride dihydrate (200 mg) were firstly dispersed in the mixture of EtOH (5 mL) and water (5 mL). After stirring for 24 h, the resulting sample was centrifuged and dried in a vacuum oven at 60 °C for 24 h. The product was then thoroughly ground and transferred to an aluminum oxide ceramic boat in a tube furnace, followed by heating at 550 °C with a ramping rate of 8 $°C\cdot min^{-1}$ under 5 % H_2/Ar atmosphere for 4 h. After naturally cooled down to the room temperature and ground, the final product of Cu-C_3N_4 was collected.

Cu-graphene was synthesized following the reported procedure[10]. First of all, graphene (100 mg) was dispersed in the mixture of EtOH and aniline (1 mL) and stirred for 5 h at room temperature. The aniline-stacked graphene was collected by centrifugation and washed with ethanol for several times. Next, the obtained aniline-stacked graphene and $CuCl_2$ (13.4 mg) were dispersed uniformly in EtOH solution (50 mL) with vigorous stirring for 5 h. After filtering and reducing by $NaBH_4$ (200 mg), the final product of Cu-Graphene was obtained by calcining at 400 °C for 2 h with a ramping rate of 2 $°C\cdot min^{-1}$.

The as-obtained C_3N_4 (50 mg) was dispersed in DMF (49 mL) with the assistance of ultrasonication for 15 min. To this suspension, a pre-determined concentration (1, 2, and 3 mg mL^{-1}) of FePc (1 mL) was added dropwise, followed by ultrasonication for 30 min (defined as FePc@C_3N_4). After continuous stirring at room temperature for 24 h, the precipitates were collected by centrifugation and washed with DMF and ethanol several times until the supernatant became colorless. Then, the as-obtained product was washed with deionized water, collected by filtration, and freeze-dried overnight to yield the final product of FePc@C_3N_4. For the preparation of FePc@Cu-C_3N_4, the same procedure was used, replacing C_3N_4 with Cu-C_3N_4 (50 mg). FePc@Graphene was also prepared following the same procedure, replacing C_3N_4 with graphene (50 mg). For the preparation of FePc@Cu-Graphene, the same procedure was employed, replacing C_3N_4 with Cu-graphene (50 mg).

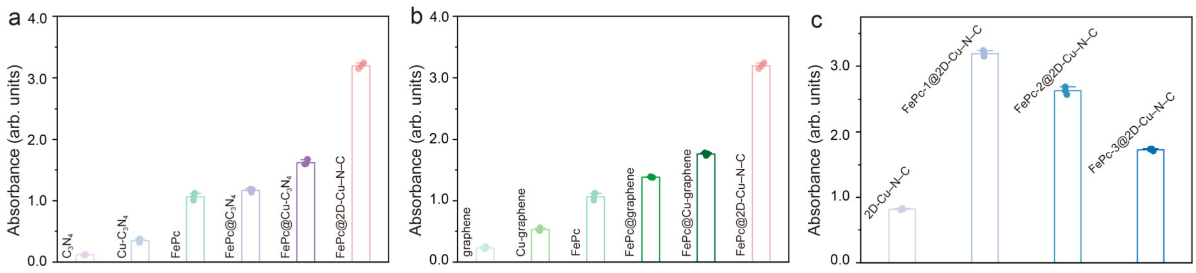

Supplementary Fig. 30. a–c, OXD-like performances of C_3N_4, FePc, FePc@C_3N_4, Cu-C_3N_4, and FePc@Cu-C_3N_4 (**a**), graphene, Cu-graphene, FePc@graphene, FePc@Cu-graphene, and FePc@2D-Cu–N–C (**b**), and FePc@2D-Cu–N–C loaded with different amounts of FePc (**c**). n = 3 independent experiments and all data are presented as mean values ± SD. Source data are provided as a Source Data file.

Supplementary Note 5:

Firstly, different N types contribute negligible OXD-like performance and are not the main effect for engineering high-performance OXD-like nanozymes (**Supplementary Fig. 28**). Upon the replacement of 2D-Cu–N–C support into C_3N_4, Cu-C_3N_4, graphene, and Cu-graphene, the corresponding OXD-like performance dramatically declines, verifying the critical role of the 2D-Cu–N–C support (**Supplementary Fig. 29 to Fig. 30**). The moderate amount of FePc molecules with uniform distribution is also crucial for the synergistic interaction between the single-atom Cu center and the axial FePc to jointly promote the OXD-like activity of FePc@2D-Cu–N–C (**Supplementary Fig. 30c**).

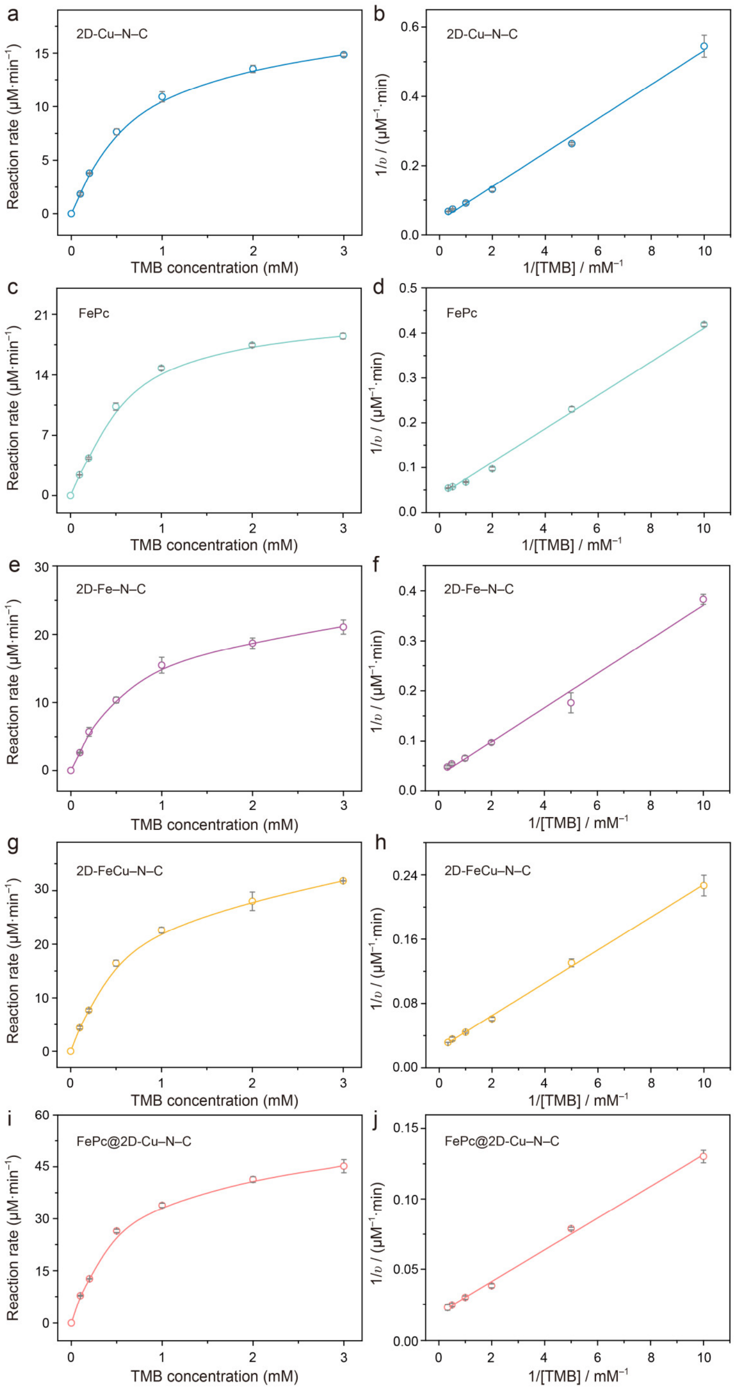

Supplementary Fig. 31. a–j, Steady-state kinetic assays of (**a–b**) 2D-Cu–N–C, (**c–d**) FePc, (**e–f**) 2D-Fe–N–C, (**g–h**) 2D-FeCu–N–C, and (**i–j**) FePc@2D-Cu–N–C. (**a, c, e, g**, and **i**) Michaelis–Menten curves with varying TMB concentration and (**b, d, f, h**, and **j**) the corresponding Lineweaver–Burk plots. *n* = 3 independent experiments and all data are presented as mean values ± SD. Source data are provided as a Source Data file.

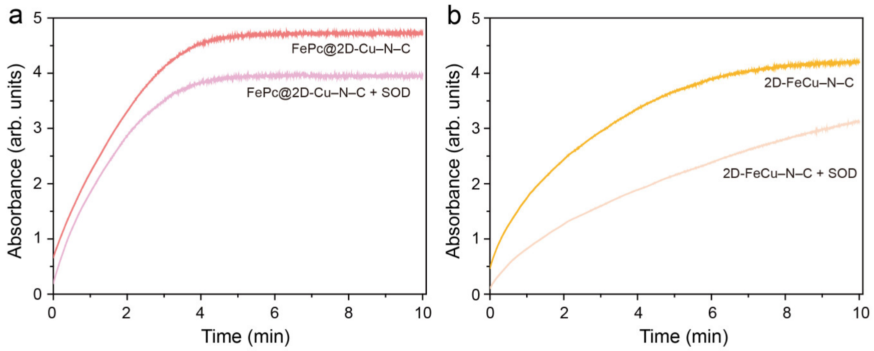

Supplementary Fig. 32. a–b, Time-dependency profiles of TMB colorimetric reaction at 652 nm catalyzed by FePc@2D-Cu–N–C (**a**) and 2D-FeCu–N–C (**b**) with and without SOD as $\bullet O_2^-$ trapping agent. Source data are provided as a Source Data file.

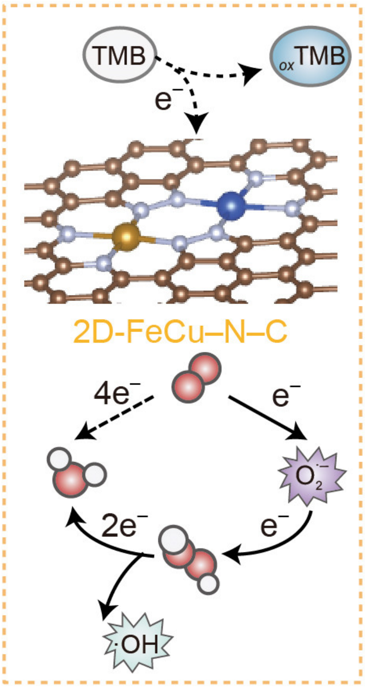

Supplementary Fig. 33. Schematic diagram illustrating the OXD-like properties of 2D-FeCu–N–C.

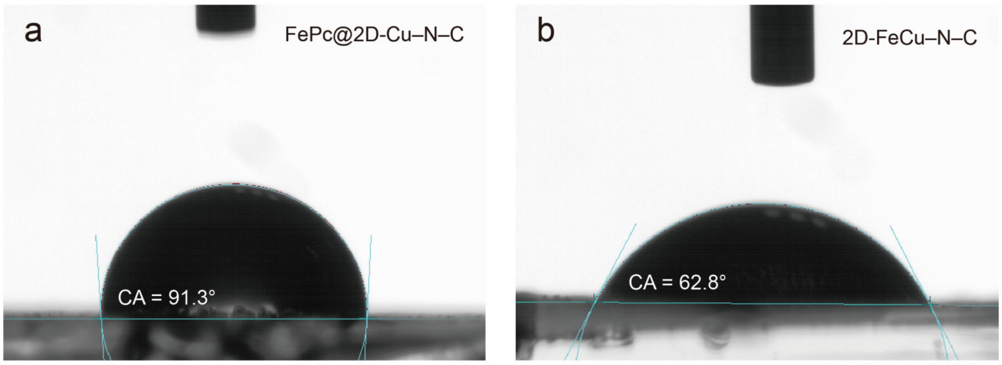

Supplementary Fig. 34. a–b, Contact angle (CA) images of FePc@2D-Cu–N–C (**a**) and 2D-FeCu–N–C (**b**).

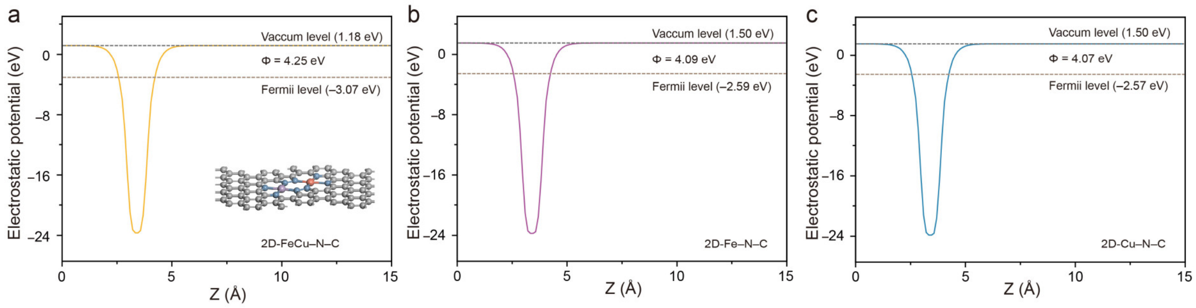

Supplementary Fig. 35. a–c, Calculated electrostatic potentials and the model structures of 2D-FeCu–N–C (**a**), 2D-Fe–N–C (**b**), and 2D-Cu–N–C (**c**). Source data are provided as a Source Data file.

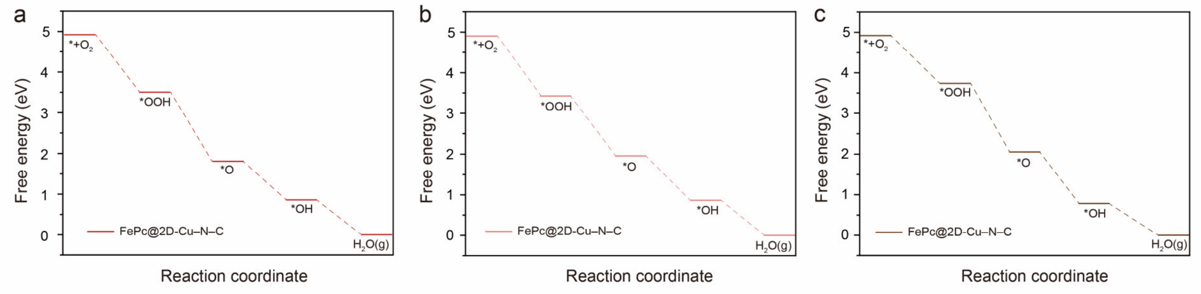

Supplementary Fig. 36. a–c, Free-energy diagrams of the OXD-like mechanism of FePc@2D-Cu–N–C using PBE0 functionals (**a**), PBE functionals (**b**), and and rPBE functionals **(c)**. Source data are provided as a Source Data file.

Supplementary Note 6:

To ascertain the accuracy of computations of the PBE functional, the more reliable revised self-interaction corrected hybrid functionals (PBE0) and revised Perdew–Burke–Emzerhof (rPBE) were also used as comparsion[11]. The result indicates that there are only minor differences between the results of PBE, PBE0, and rPBE functionals, which verifies the validity of the conclusions in this work (**Supplementary Fig. 36**).

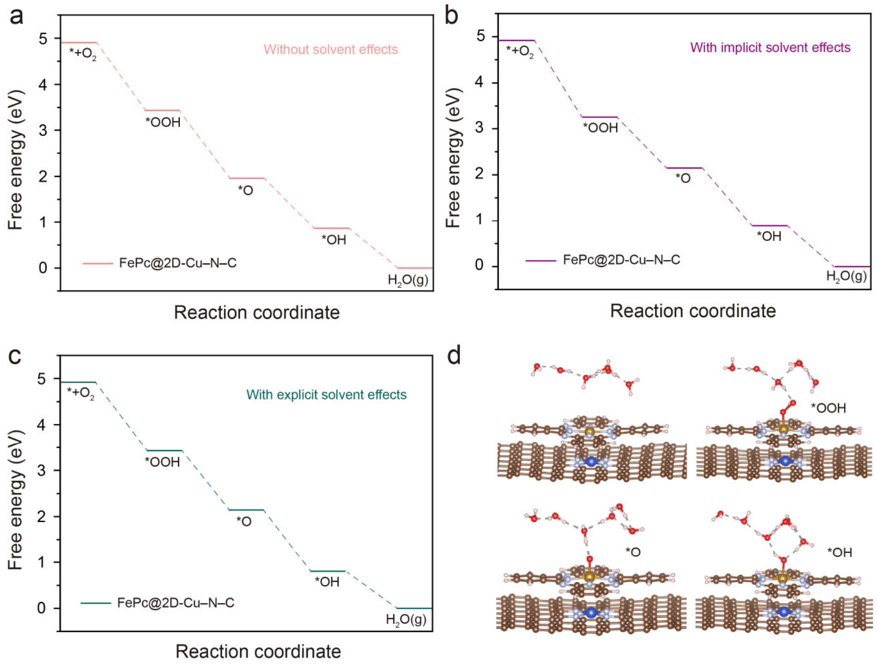

Supplementary Fig. 37. a–d, Free-energy diagrams of the OXD-like mechanism of FePc@2D-Cu–N–C without solvent effects (**a**) with implicit solvent effects **(b)**, with explicit solvent effects **(c)**, and the corresponding models with solvent effects **(d)**. Source data are provided as a Source Data file.

Supplementary Note 7:

The solvent effect was also taken into consideration by employing both the implicit and the explicit solvation models. A minimal energy difference (< 0.05 eV) is observed when considering the solvation effects, suggesting the negligible influence on the OXD-like enzymatic reaction (**Supplementary Fig. 37**).

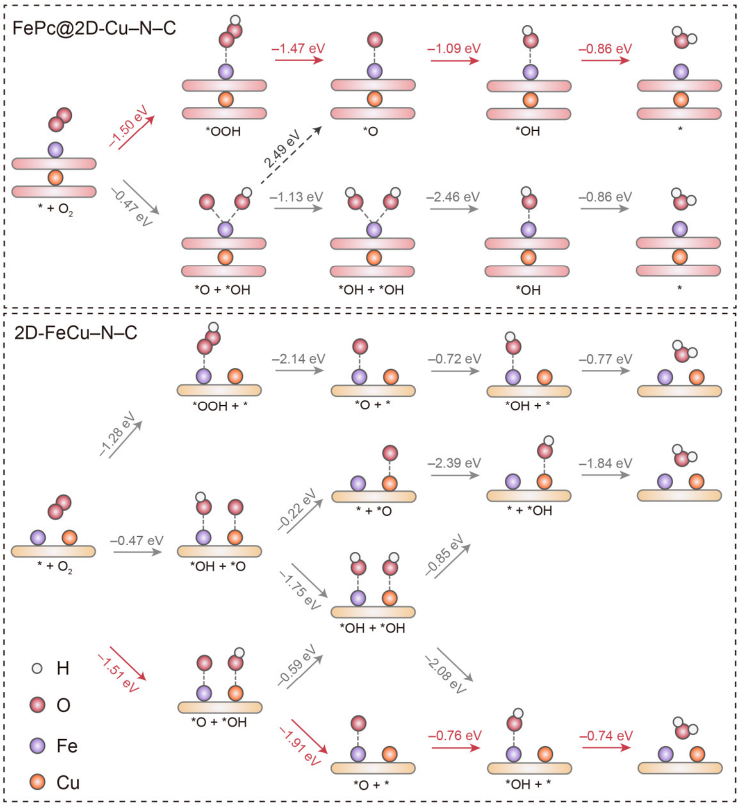

Supplementary Fig. 38. The OXD-like reaction pathway with the corresponding structure and adsorbed intermediates of FePc@2D-Cu–N–C and 2D-FeCu–N–C.

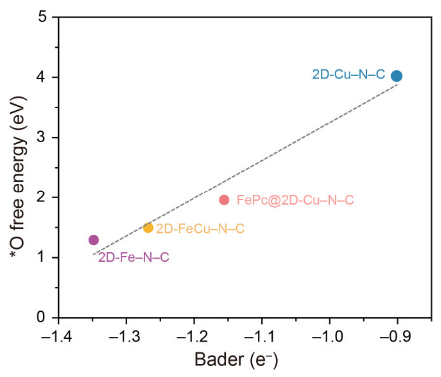

Supplementary Fig. 39. Relationship between charges of active sites and the free energy of *O absorbed on different model surfaces. Source data are provided as a Source Data file.

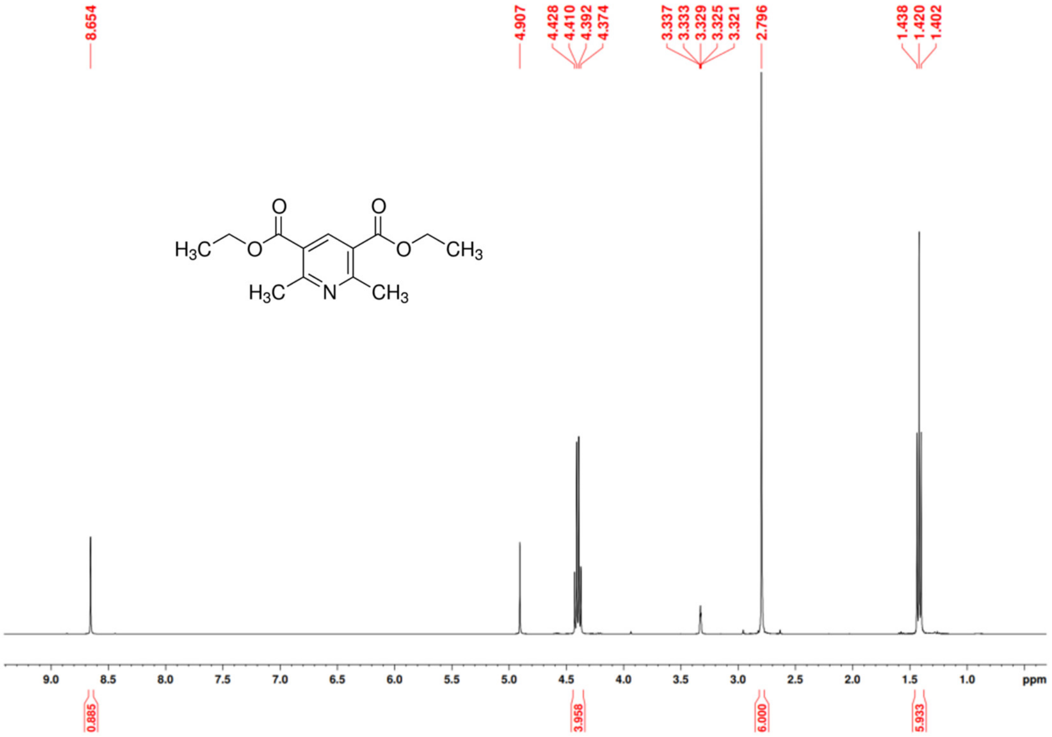

Supplementary Fig. 40. ^{1}H NMR spectrum (400 MHz, CD_3OD) of the 1,4-DHP oxidation product (DDPD) by FePc@2D-Cu–N–C. Source data are provided as a Source Data file.

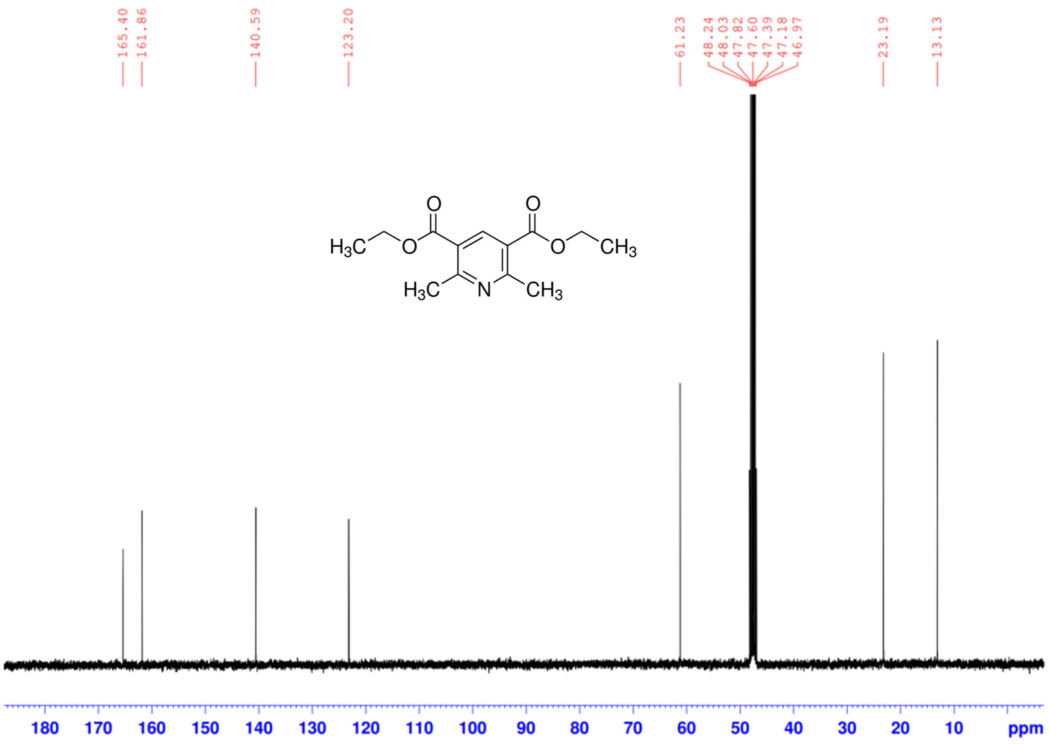

Supplementary Fig. 41. ^{13}C NMR spectrum (400 MHz, CD_3OD) of the 1,4-DHP oxidation product (DDPD) by FePc@2D-Cu–N–C. Source data are provided as a Source Data file.

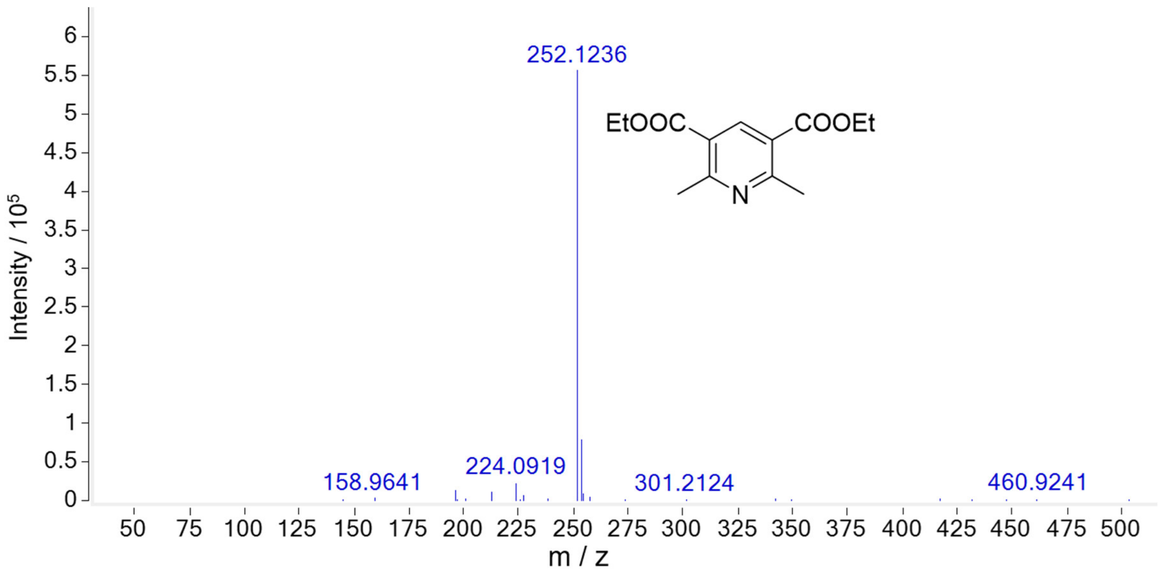

Supplementary Fig. 42. Electrospray ionization (ESI) mass spectrum in the positive ion mode for monitoring the 1,4-DHP oxidation product (DDPD) by FePc@2D-Cu–N–C. Source data are provided as a Source Data file.

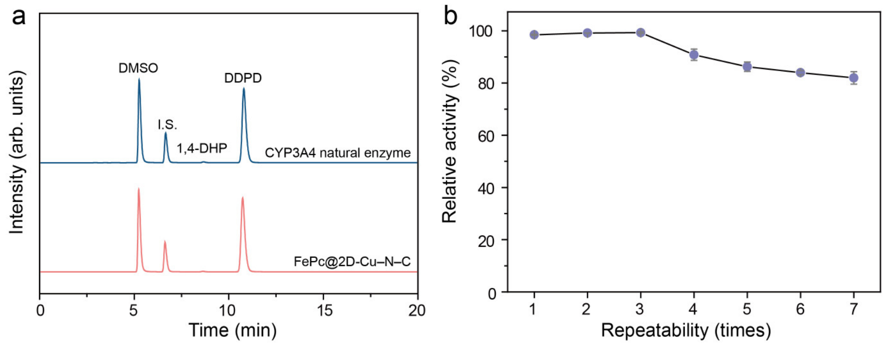

Supplementary Fig. 43. a–b, HPLC chromatograms of 1,4-DHP oxidation product (DDPD) using FePc@2D-Cu–N–C and CYP3A4 natural enzyme **(a)**. Recovery and recyclability of FePc@2D-Cu–N–C for the oxidation of 1,4-DHP **(b)** (n = 3 independent experiments and all data are presented as mean values ± SD). Source data are provided as a Source Data file.

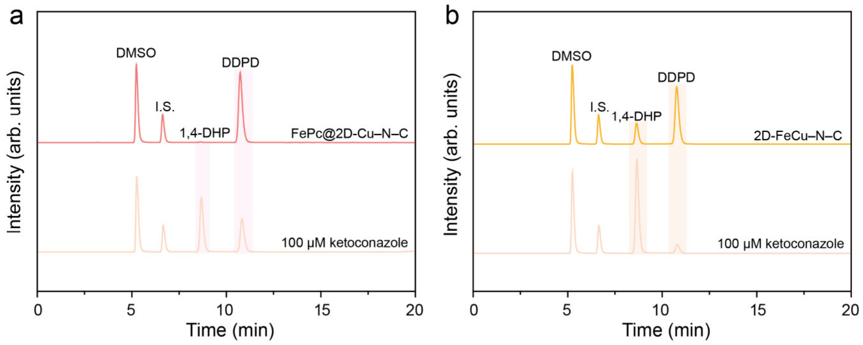

Supplementary Fig. 44. a–b, HPLC chromatograms of 1,4-DHP oxidation product (DDPD) using FePc@2D-Cu–N–C (**a**) and 2D-FeCu–N–C (**b**) as the CYP3A4-mimic with and without ketoconazole. Source data are provided as a Source Data file.

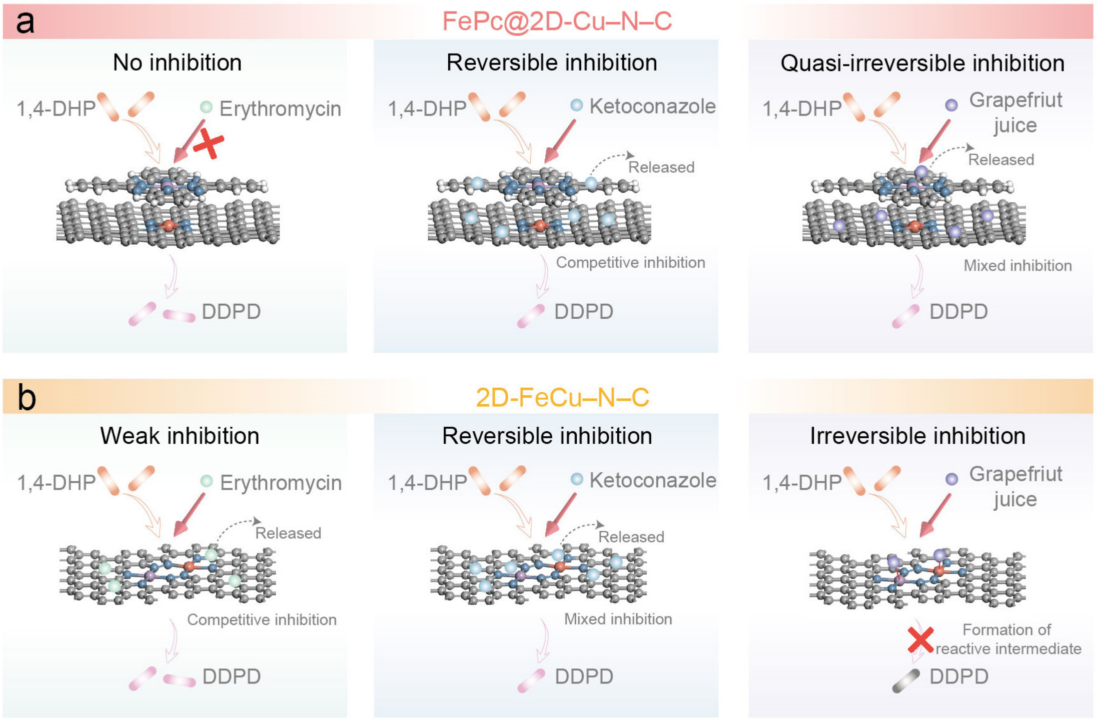

Supplementary Fig. 45. a–b, Possible mechanisms of CYP3A4-mimicking inhibition based on FePc@2D-Cu–N–C (**a**) and 2D-FeCu–N–C (**b**) as the CYP3A4-mimic.

Supplementary Tables

Supplementary Table 1. Cu and Fe contents in 2D-Cu–N–C, 2D-Fe–N–C, 2D-FeCu–N–C, and FePc@2D-Cu–N–C catalysts based on elemental analysis and ICP-OES results.

Sample	Cu content (wt.%)	Fe content (wt.%)
**2D-Cu–N–C**	5.83	-
**2D-Fe–N–C**	-	6.98
**2D-FeCu–N–C**	3.89	5.57
**FePc@2D-Cu–N–C**	4.37	5.13

Supplementary Table 2. Summary of Fe K-edge EXAFS curves fitting parameters.

Sample	Path	Coordination Number, CN	Bond Length, R (Å)	Bond Disorder, $\sigma^{2[a]}$ ($\times10^{-2}$ Å^2)	ΔE_0[b] (eV)	*R* Factor[c]
**Fe foil**	Fe–Fe	8*	2.47 ± 0.01	0.6 ± 0.1	6.0 ± 1.0	0.018
	Fe–Fe	6*	2.85 ± 0.01	0.6 ± 0.1		
	Fe–Fe	12*	4.06 ± 0.01	0.9 ± 0.2		
	Fe–Fe	24*	4.74 ± 0.01	0.4 ± 0.1		
**FePc@2D-Cu–N–C**	Fe–N	4.0	1.99 ± 0.01	0.6 ± 0.1	6.0±1.0	0.004
	Fe–C	8.0	3.04 ± 0.01	0.4 ± 0.1		
	Fe–N	2.0	3.30 ± 0.01	0.4 ± 0.1		
	Fe–Cu	1.0	2.97 ± 0.01	0.4 ± 0.1		
**2D-FeCu–N–C**	Fe–N	4.0	2.01 ± 0.02	0.5 ± 0.1	4.0 ± 2.0	0.024
	Fe–C	2.0	3.14 ± 0.07	1.1 ± 0.9		
	Fe–C	2.0	3.37 ± 0.08	0.7 ± 0.4		
	Fe–Cu	1.0	4.06 ± 0.08	1.1 ± 0.9		

[a] σ^2: Debye-Waller factor. [b] ΔE: the inner potential correction. [c] *R* factor: goodness of fit.

*The experimental EXAFS fit of the metal foil by fixing CN as the known crystallographic value.

Supplementary Table 3. Summary of Cu K-edge EXAFS curves fitting parameters.

Sample	Path	Coordination Number, CN	Bond Length, R (Å)	Bond Disorder, $\sigma^{2[a]}$ ($\times 10^{-2}$ Å^2)	ΔE_0[b] (eV)	*R* Factor[c]
**Cu foil**	Cu–Cu	12*	2.54 ± 0.01	0.9 ± 0.1	4.0 ± 1.0	0.014
	Cu–Cu	6*	3.58 ± 0.02	1.2 ± 0.2		
	Cu–Cu	24*	4.45 ± 0.01	1.1 ± 0.1		
**FePc@2D-Cu–N–C**	Cu–N	4.0	1.98 ± 0.02	0.7 ± 0.3	−5.0 ± 3.0	0.010
	Cu–Fe	1.0	2.97 ± 0.02	0.9 ± 0.3	−2.0 ± 3.0	
**2D-FeCu–N–C**	Cu–N	4.0	1.95 ± 0.02	0.5 ± 0.2	−4.0 ± 3.0	0.018
**2D-Cu–N–C**	Cu–N	4.0	1.95 ± 0.02	0.2 ± 0.1	−5.0 ± 2.0	0.010

[a] σ^2: Debye-Waller factor. [b] ΔE: the inner potential correction. [c] *R* factor: goodness of fit.

*The experimental EXAFS fit of the metal foil by fixing CN as the known crystallographic value.

Supplementary Table 4. Summary of the paramters for ^{57}Fe Mössbauer spectroscopy of FePc@2D-Cu–N–C.

Fe species	IS (mm s^{-1})	QS (mm s^{-1})	Content (%)
**D1**	0.188	1.04	51.53
**D2**	0.36	0.64	34.36
**D3**	0.178	2.65	14.11

Supplementary Table 5. Summary of the apparent Michaelis-Menten constant (K_{m}), maximum reaction rate ($V_{max.}$), catalytic constant ($k_{cat.}$), and catalytic efficiency ($k_{cat.}/K_{m}$) of various catalysts as oxidase mimetic.

**Catalyst**	K_{m} **(mM)**	$V_{max.}$ **(μM·min^{-1})**	k_{cat} **(s^{-1})**	k_{cat}/K_{m} **(10^{3}·M^{-1}·s^{-1})**
**FePc**	1.156	30.03	0.70	0.60
**2D-Cu–N–C**	1.192	24.27	1.10	0.92
**2D-Fe–N–C**	1.144	33.56	1.12	0.98
**2D-FeCu–N–C**	0.84	41.15	1.72	2.05
**FePc@2D-Cu–N–C**	0.604	53.48	2.43	4.02

Supplementary Table 6. The computed free energy changes (ΔG, eV) of each possible elementary step for the overall OXD-like enzymatic reaction pathway on FePc@2D-Cu–N–C. The marked red step represents its higher selectivity.

**Elementary step**	**Free energy change (ΔG)**
$* + O_2 + H^+ \rightarrow *OOH$	–1.50
$* + O_2 + H^+ \rightarrow *O*OH$	–0.47
$*OOH + H^+ \rightarrow *O + H_2O$	–1.47
$*O + H^+ \rightarrow *OH$	–1.09
$*OH + H^+ \rightarrow * + H_2O$	–0.86

Supplementary Table 7. The computed free energy changes (ΔG, eV) of each possible elementary step for the overall OXD-like enzymatic reaction pathway on 2D-FeCu–N–C. The marked red step represents its higher selectivity.

**Elementary step**	**Free energy change (ΔG)**
$* + O_2 + H^+ \rightarrow *OOH$	–1.28
$* + O_2 + H^+ \rightarrow *OH*O$	–0.47
$* + O_2 + H^+ \rightarrow *O*OH$	–1.51
$*O*OH + H^+ \rightarrow *O + H_2O$	–1.91
$*O*OH + H^+ \rightarrow *OHOH$	–0.59
$*O + H^+ \rightarrow *OH$	–0.76
$*OH + H^+ \rightarrow * + H_2O$	–0.74

Supplementary References

1. Newville M. IFEFFIT: interactive XAFS analysis and FEFF fitting. *J. Synchrotron Radiat.* **8**, 322-324 (2001).
2. Ravel B, Newville M. ATHENA, ARTEMIS, HEPHAESTUS: data analysis for X-ray absorption spectroscopy using IFEFFIT. *J. Synchrotron Radiat.* **12**, 537-541 (2005).
3. Joly Y, Ramos AY, Bunău O. Finite-difference method for the calculation of X-ray spectroscopies. *Int. Tables Crystallogr.* **1**, 1-7 (2022).
4. Bunău O, Ramos A, Joly Y. The FDMNES code. *Int. Tables Crystallogr.* **1**, 1-6 (2021).
5. Peng L*, et al.* Mesopore-rich Fe-N-C catalyst with FeN_4-O-NC single-atom sites delivers remarkable oxygen reduction reaction performance in alkaline media. *Adv. Mater.* **34**, 2202544 (2022).
6. Ren W*, et al.* Confinement of ionic liquids at single-Ni-sites boost electroreduction of CO_2 in aqueous electrolytes. *ACS Catal.* **10**, 13171-13178 (2020).
7. Jia Q*, et al.* Experimental observation of redox-induced Fe−N switching behavior as a determinant role for oxygen reduction activity. *ACS Nano* **9**, 12496-12505 (2015).
8. Zhuang Z*, et al.* Continuous modulation of electrocatalytic oxygen reduction activities of single-atom catalysts through p-n junction rectification. *Angew. Chem. Int. Ed.* **62**, e202212335 (2023).
9. Yang T*, et al.* Coordination tailoring of Cu single sites on C_3N_4 realizes selective CO_2 hydrogenation at low temperature. *Nat. Commun.* **12**, 6022 (2021).
10. Zhang Y*, et al.* N-doped graphene supported Cu single atoms: highly efficient recyclable catalyst for enhanced C–N coupling reactions. *ACS Nano* **16**, 1142-1149 (2022).
11. Patel AM, Ringe S, Siahrostami S, Bajdich M, Nørskov JK, Kulkarni AR. Theoretical approaches to describing the oxygen reduction reaction activity of single-atom catalysts. *J. Phys. Chem. C* **122**, 29307-29318 (2018).
