# Supplementary material for: Spatial engineering of single-atom Fe adjacent to Cu-assisted nanozymes for biomimetic O2 activation
Source: Nat Commun. 2024 Mar 12;15:2239. doi: 10.1038/s41467-024-46528-w (PMC10933453; doi:10.1038/s41467-024-46528-w)
Supplement: Supplementary file 2 — Reporting Summary [file 41467_2024_46528_MOESM2_ESM.pdf]

Corresponding author(s): Kwok-Yin Wong

Last updated by author(s): Feb, 23 2024

Reporting Summary

Nature Portfolio wishes to improve the reproducibility of the work that we publish. This form provides structure for consistency and transparency in reporting. For further information on Nature Portfolio policies, see our Editorial Policies and the Editorial Policy Checklist.

Statistics

For all statistical analyses, confirm that the following items are present in the figure legend, table legend, main text, or Methods section.

n/a	Confirmed	
☐	☒	The exact sample size (*n*) for each experimental group/condition, given as a discrete number and unit of measurement
☐	☒	A statement on whether measurements were taken from distinct samples or whether the same sample was measured repeatedly
☒	☐	The statistical test(s) used AND whether they are one- or two-sided<br>*Only common tests should be described solely by name; describe more complex techniques in the Methods section.*
☒	☐	A description of all covariates tested
☒	☐	A description of any assumptions or corrections, such as tests of normality and adjustment for multiple comparisons
☐	☒	A full description of the statistical parameters including central tendency (e.g. means) or other basic estimates (e.g. regression coefficient) AND variation (e.g. standard deviation) or associated estimates of uncertainty (e.g. confidence intervals)
☒	☐	For null hypothesis testing, the test statistic (e.g. *F*, *t*, *r*) with confidence intervals, effect sizes, degrees of freedom and *P* value noted<br>*Give P values as exact values whenever suitable.*
☒	☐	For Bayesian analysis, information on the choice of priors and Markov chain Monte Carlo settings
☒	☐	For hierarchical and complex designs, identification of the appropriate level for tests and full reporting of outcomes
☒	☐	Estimates of effect sizes (e.g. Cohen's *d*, Pearson's *r*), indicating how they were calculated

Our web collection on statistics for biologists contains articles on many of the points above.

Software and code

Policy information about availability of computer code

Data collection	The data were collected on Rigaku SmartLab 9 kW Advance (XRD), Hitachi-SU8010 (SEM), Tescan VEGA3 (SEM), JEOL JEM-2100F (TEM, SAED, EDX), Thermo Fisher FEI Themis Z (HAADF-STEM), Thermo Scientific™ Spectra 300 S/TEM (iDPC-STEM, EELS), Bruker Dimension Icon (AFM), (Renishaw (Raman spectra), Agilent 710 Series (ICP-OES), Thermo Fisher Scientific ESCALAB-250 (XPS and UPS), Thermo Fisher Scientific Nexus 470 (FT-IR), Malvern Panalytical Zetasizer Nano ZS and BeNano 180 Zeta (Zeta potential), DataPhysics Instruments OCA 25 (Contact angle images), Bruker EMXplus-6/1 (EPR), Cary 4000 UV–vis spectrophotometer (UV–vis), BL8C beamline (Pohang Light Source, XAS), Quantum Design (temperature-dependent magnetic susceptibility),Agilent 1200 series (HPLC), ADVANCE III 400, Bruker (NMR), and Thermo Finnigan MAT Bremen (ESI-MS).
Data analysis	In this study, Origin 2018, NanoScope Analysis 1.7, IFEFFIT-1.2.10, XPSPEAK41, VASP.5.4.4, Gatan Microscopy Suite 3, and GraphPad Prism 9 were used to analyze the data.

For manuscripts utilizing custom algorithms or software that are central to the research but not yet described in published literature, software must be made available to editors and reviewers. We strongly encourage code deposition in a community repository (e.g. GitHub). See the Nature Portfolio guidelines for submitting code & software for further information.

Data

Policy information about availability of data

All manuscripts must include a data availability statement. This statement should provide the following information, where applicable:

- Accession codes, unique identifiers, or web links for publicly available datasets
- A description of any restrictions on data availability
- For clinical datasets or third party data, please ensure that the statement adheres to our policy

All the data supporting the findings of this study are available within the article, source data, and its supplementary information files. Source data are provided as a Source Data file. This study uses publicly available data from the Protein Data Bank (PDB) under accession codes: 1OCR.

Research involving human participants, their data, or biological material

Policy information about studies with human participants or human data. See also policy information about and sexual orientation and race, ethnicity and racism.

Reporting on sex and gender	N/A
Reporting on race, ethnicity, or other socially relevant groupings	N/A
Population characteristics	N/A
Recruitment	N/A
Ethics oversight	N/A

Note that full information on the approval of the study protocol must also be provided in the manuscript.

Field-specific reporting

Please select the one below that is the best fit for your research. If you are not sure, read the appropriate sections before making your selection.

☒ Life sciences ☐ Behavioural & social sciences ☐ Ecological, evolutionary & environmental sciences

For a reference copy of the document with all sections, see

Life sciences study design

All studies must disclose on these points even when the disclosure is negative.

Sample size	No statistical methods were applied to predetermine sample sizes. Sample sizes were determined empirically from previous experimental experience with similar assays or from sizes generally employed in the field. For material synthesis and enzyme-like activity test, each group involved more than three times evaluation processes (**images from three independent samples, test from three independent experiments**).
Data exclusions	No data was excluded from the analysis.
Replication	All experiments were repeated a minimum of two times to confirm the repeatability of the findings. All repeats of experiments were successful.
Randomization	All samples were randomly allocated into experimental groups.
Blinding	All the characterizations were conducted with the sample random grouped, and the investigators were blinded during data collection and analysis.

Reporting for specific materials, systems and methods

We require information from authors about some types of materials, experimental systems and methods used in many studies. Here, indicate whether each material, system or method listed is relevant to your study. If you are not sure if a list item applies to your research, read the appropriate section before selecting a response.

Materials & experimental systems

n/a	Involved in the study
☒	☐ Antibodies
☒	☐ Eukaryotic cell lines
☒	☐ Palaeontology and archaeology
☒	☐ Animals and other organisms
☒	☐ Clinical data
☒	☐ Dual use research of concern
☒	☐ Plants

Methods

n/a	Involved in the study
☒	☐ ChIP-seq
☒	☐ Flow cytometry
☒	☐ MRI-based neuroimaging

Plants

Seed stocks	N/A
Novel plant genotypes	N/A
Authentication	*Describe any authentication procedures for each seed stock used or novel genotype generated. Describe any experiments used to assess the effect of a mutation and, where applicable, how potential secondary effects (e.g. second site T-DNA insertions, mosiacism, off-target gene editing) were examined.*
